# Comparative study of bacteriological culture and real-time fluorescence quantitative PCR (RT-PCR) and multiplex PCR-based reverse line blot (mPCR/RLB) hybridization assay in the diagnosis of bacterial neonatal meningitis

**DOI:** 10.1186/1471-2431-14-224

**Published:** 2014-09-08

**Authors:** Yajuan Wang, Gaili Guo, Huixin Wang, Xuefang Yang, Fang Shao, Caiyun Yang, Wei Gao, Zhujun Shao, Jinjing Zhang, Jie Luo, Yonghong Yang, Fanrong Kong, Bingqing Zhu

**Affiliations:** Neonatal Center, Beijing Children’s Hospital, Capital Medical University, Beijing, 100045 China; Laboratory of Microbiology and Immunology, Beijing Children’s Hospital, Capital Medical University, Beijing, 100045 China; Chinese Center for Disease Control and Prevention, Beijing, China; Center for Infectious Diseases and Microbiology (CIDM), Institute of Clinical, Pathology and Medical Research (ICPMR), Westmead, New South Wales Australia

**Keywords:** Neonate, Bacterial meningitis, Bacterial pathogens identification, Multiplex real-time fluorescence quantitative PCR (RT-PCR), Multiplex PCR based-reverse line blot hybridization (mPCR/RLB) assay, Bacteria culture

## Abstract

**Background:**

Bacterial meningitis is more common in the neonatal period than any other time in life; however, it is still a challenge for the evidence based diagnosis. Strategy for identification of neonatal bacterial meningitis pathogens is presented by evaluating three different available methods to establish evidence-based diagnosis for neonatal bacterial meningitis.

**Methods:**

The cerebrospinal fluid samples from 56 neonates diagnosed as bacterial meningitis in 2009 in Beijing Children’s Hospital were analyzed in the study. Two PCR based molecular assays, real-time fluorescence quantitative PCR (RT-PCR) and multiplex PCR based-reverse line blot hybridization (mPCR/RLB), were used to assess 7 common neonatal meningitis bacterial pathongens, including *Escherichia coli*, *Staphylococcus aureus*, *Listerisa monocytogen*es, *Neisseria meningitidis*, *Haemophilus influenzae*, *Streptococcus pneumoniae*, and *Streptococcus agalactiae*. The findings in examinations of two assays were compared with the results obtained bacterial culture tests.

**Results:**

Bacterial meningitis was identified in five cases (9%) by CSF cultures, 25 (45%) by RT-PCR and 16 (29%) by mPCR/RLB. One strain of *S. epidermidis* and one of *E. faecalis* were identified using mPCR/RLB but not by RT-PCR. In contrast, cultures identified one strain of *S. pneumoniae* which was missed by both PCR assays. Overall, the bacterial pathogens in 28 cases were identified with these three methods. Both RT-PCR and mPCR/RLB assays were more sensitive than bacterial culture, (*p* < 0.05).

**Conclusion:**

Our study confirmed that both RT-PCR and mPCR/RLB assays have better sensitivity than bacterial culture. They are capable of detecting the pathogens in CSF samples with negative culture results.

## Background

Bacterial meningitis is more common in the neonatal period than any other time in life [1–4]. A recent review on neonatal infections reports that the incidence of meningitis ranges from 0.8 to 6.1 cases per 1,000 live newborns [5]. The World Health Organization (WHO) estimates that there are approximately 5 million neonatal deaths a year, and the fatality rate of neonatal meningitis is as high as 50% [6, 7]. The overwhelming majority (98%) of fatal cases of neonatal meningitis occurs in developing countries. Moreover, 21% to 50% of the survivors show neurological sequelae with hydrocephalus, blindness, hearing loss, paralysis, and mental retardation [4].

Signs and symptoms of neonatal bacterial meningitis may be subtle, nonspecific, vague, and atypical. A high index of suspicion is therefore needed to initiate investigations. Further, the identification of specific organisms and their sensitivity is important for the judicious and rational use of antimicrobial agents. No single cerebrospinal fluid (CSF) value can be used to exclude meningitis, and peripheral WBC counts are poor predictors of neonatal meningitis [8]. Currently, few techniques for the rapid diagnosis of neonatal bacterial meningitis are available, and the commercial molecular tests are generally too expensive for developing countries. The bacterial culture remains as the gold standard though there is low recovery rate of pathogens.

Non-culture methods, such as multiplex real-time fluorescence quantitative PCR (RT-PCR) and multiplex polymerase chain reaction (mPCR)-based reverse line blot (RLB) hybridization assays are reliable and accurate tests which could increase the diagnostic yield of bacterial meningitis [9–13]. Both RT-PCR and mPCR/RLB assays have been used as excellent tool in epidemiologic studies [14, 15]. The RT-PCR assay has characteristic of high sensitivity in CSF [10]; the mPCR/RLB approach is suited for the batched simultaneous analysis of large numbers of isolates [15].

The aim of this study was to evaluate the optimal strategy for identification of bacterial pathogens in neonatal bacterial meningitis based on the three methods including RT-PCR, mPCR/RLB and CSF bacterial culture.

## Methods

### Patient **s**election

This is a cross sectional design study. This study enrolled newborns with bacterial meningitis aged between 0 to 28 days admitted to the neonatal department in Beijing Children’s Hospital, affiliated to Capital Medical University in 2009. The bacterial meningitis was diagnosed based on clinical presentation, abnormal laboratory tests and CSF culture [16]. The diagnostic laboratory criteria for bacterial meningitis included the following: presence of >20 leukocytes/mm^3^, predominance of neutrophils; protein concentration in cerebrospinal fluid >150 mg/dL; hypoglycorrhachia <1.1-2.2 mmol/L or <50%-75% of the concomitant blood glucose concentration; identification of bacteria on microscopy and/or culture of eliminate CSF. The clinical manifestations suggestive of neonatal meningitis included lethargy, vomiting, convulsions, irritability, refuses to feed, tremors and bulging fontanels. The exclusion criteria consisted of central nervous system malformations, meningitis after cranio-encephalic trauma, and viral or fungal meningitis.

Our study was agreed by the Research Ethical Review Committee, Beijing Children’s Hospital Affiliated to Capital Medical University. The written consent informs were obtained from the parents of all participants.

### CSF samples

The initial CSF samples were used for analysis, which were collected by an experienced physician under aseptic condition. The volume, turbidity, cells, protein and glucose concentration of CSF samples were measured. 100 μl of CSF was inoculated onto each bacterial culture plate. The remaining samples were placed into two sterile tubes, (0.5-1 ml), and stored at −70°C.

### Bacterial isolates

Reference strains of 7 bacterial species - *Escherichia coli*, *Staphylococcus aureus*, *Listerisa monocytogen*es, *Neisseria meningitidis*, *Haemophilus influenzae*, *Streptococcus pneumoniae*, and *Streptococcus agalactiae*, as listed in Table 1, were used to develop the assays. RT-PCR and mPCR/RLB were developed to detect the target genes of the specific pathogens from prior clinical isolates. All clinical isolates were identified according to conventional standard methods.

**Table 1:** **Bacterial species and isolates used to develop and evaluate the RT-PCR, mPCR/RLB and DNA detection limits for each species**

Species	Strain ID number	RT-PCR			mPCR/RLB			Clinical isolates (No)
		Target gene	Detection limit	Genome copies/μL	Target gene	Detection limit	Genome copies/μL	
*E. coli*	ATCC 25922	16S rRNA	2 fg	0.3	16S rRNA	500 fg	90	6
*S. aureus*	ATCC 25923	*femA*	200 fg	63	*nuc*	500 fg	160	9
*L. monocytogenes*	ATCC 19112	*hly*	200 fg	62	*hly*	50 fg	15	2
*N. meningitides*	ATCC 29019	*ctrA*	20 fg	8	*ctrA*	500 fg	200	2
*S. pneumonia*e	SSI serotype 14	*lytA*	200 fg	90	*ply*	500 fg	230	7
*H. influenza*	ATCC 10211	*bexA*	20 fg	10	*gyrB*	500 fg	255	10
*S. agalactiae*	ATCC A2	*cfb*	200 fg	91	*cfb*	500 fg	228	5

Abbreviations: ATCC – American Type Culture Collection.

Genome copies/μl = concentration (ng/μl) × 6.02 × 10^23^ × 10^−9^/660× whole genome nucleotide number. The number of whole-genome nucleotide: *E. coli*-5498450-bp, *S. aureus*-2902619-bp, *L. monocytogenes*-2944528-bp, *N. meningitides*-2194961-bp, *S. pneumonia* ***e −***2046115-bp, *H. influenza*-1830138-bp, *S. agalactiae −*2160267-bp.

### DNA extraction

DNA extraction was performed using QIAGEN QIAamp DNA Blood mini kit (QIAGEN, Shanghai, China) following the product instruction. An aliquot of 200 μL of CSF was processed and the DNA was eluted in 100 μL of TE buffer.

### RT-PCR

A RT-PCR assay on Mx3000P QPCR Systems (Stratagene, USA) was used to identify seven common pathogens that cause neonatal bacterial meningitis.

#### Primer and probe design

Species-specific primers and probes were designed, followed previously validated methods, to allow amplification [17–21]. The details of the primers and the probes in this assay are shown in Table 2.

**Table 2:** **Primers and probes used in RT-PCR assay**

Specificity	Target		Primer and probe sequence (5′- 3′)	Application product (bp)
*E.coli*	16S rRNA	F	GGGAGTAAAGTTAATACCTTTGC	204
		R	CTCAAGCTTGCCAGTATCAG	
		Probe	FAM-CGCGATCACTCCGTGCCAGCAGCCGCGGATCGCG-BHQ1	
*L.monocytogenes*	*hly*	F	CAT GGCACCACCAGC ATCT	64
		R	ATC CGCGTGTTTCTTTTCGA	
		Probe	HEX-CGCCTG CAA GTC CTA AGA CGC CA-BHQ1	
*S. aureus*	*femA*	F	TGCTGGTGGTACATCAAA	97
		R	ACGGTCAATGCCATGATTTAA	
		Probe	FAM-ATTTTGCCGGAAGTTATGCAGTGCAATG-BHQ1	
*N. meningitidis*	*ctrA*	F	TGTGTTCCGCTATACGCCATT	114
		R	GCCATATTCACACGATATACC	
		Probe	FAM-AACCTTGAGCAA“T”CCATTTATCCTGACGTTCT-SpC6 “T”-BHQ1	
*H. influenzae*	*bexA*	F	TGCGGTAGTGTTAGAAAATGGTATTATG	116
		R	GGACAAACATCACAAGCGGTTA	
		Probe	HEX-ACAAAGCGTATCAA“T”ACTACAACGAGACGCAAAAA-SpC6 “T”-BHQ	
*S. pneumoniae*	*lytA*	F	ACGCAATCTAGCAGATGAAGCA	75
		R	TCGTGCGTTTTAATTCCAGCT	
		Probe	FAM-TGCCGAAAACGCTTGATACAGGGAG-BHQ1	
*S. agalactiae*	*cfb*	F	CGCAATGAAGTCTTTAATTTTTC	260
		R	ATGATGTATCTATCTGGAACTCTAGTG	

The assays were carried out in a final 20 μL reaction volume and were performed using 2 × PCR Premix Ex *Taq* (Stratagene, USA), with 2 μL of sample extracted DNA. Forward primer, reverse primer, and probe for each gene target were mixed. The probes were labeled at the 5′ end with FAM and HEX, respectively. RT-PCR was performed at 95°C for 2 min, followed by 35–50 cycles of 95°C for 5 sec and 60-73°C for 20 sec (see Table 3 for detail).

**Table 3:** **Thermal profiles of RT-PCR**

Specificity	Thermal profiles
*N. meningitides*	95°C for 2 min, followed by 50 cycles of 95°C for 5 sec and 60°C for 20 sec
*S. aureus*	
*H. influenzae*	
*S. pneumoniae*	
*L.monocytogenes*	95°C for 2 min, followed by 50 cycles of 95°C for 5 sec and 63°C for 20 sec
*E.coli*	95°C for 30 sec, followed by 50 cycles of 95°C for 15 sec, 50°C for 30 sec and 75°C 20s
*S. agalactiae*	95°C for 30 sec, followed by 35 cycles of 94°C for 10 sec, 60°C for 15 sec and 72°C for 25s

#### Analytical sensitivity and specificity

DNA was extracted from reference strains using a DNA Miniprep Extraction kit (Sigma, St Louis, MO, USA) according to the manufacturer’s instructions, and was tested against all primer sets. The lower limit of detection (LLD) was determined using extracted DNA from one isolate each of *E. coli*, *S. aureus*, *L. monocytogenes*, *N. meningitidis*, *S. pneumoniae*, *H. influenzae*, and group B streptococci*.* DNA concentration was adjusted to 100 ng/mL from which serial ten-fold dilutions of genomic DNA were prepared in distilled water. In addition, serial ten-fold dilutions of suspensions of cultures of seven bacteria were prepared in physiological saline. Crude DNA was extracted from 1 mL of these suspensions being heated to 100°C for 10 min.

### mPCR/RLB

The mPCR/RLB assay was used to identify the seven pathogens, same as shown in Table 1.

#### Primer and probe design

Species-specific primers and probes were designed, based on a previously validated method [4]. Primers were labeled at the 5′end with biotin to allow PCR products to be detected by hybridization with a streptavidin–peroxidase substrate in the RLB assay. All probes were labeled at the 5′end with an amine group to facilitate covalent linkage to nylon membranes and to allow membranes to be stripped and reused repeatedly.

#### mPCR/RLB

The mPCR mixture containing 14 primer-pairs included 5 μL DNA extract, 0.25 μL each forward (50 umol / L) and reverse (50 μmol /L) primer, 1.25 μL dNTPs mix (2.5 mM each dNTP), 2.5 μl 10 × PCR buffer, 4.5 mM MgCl2 (final concentration), 3uL Hotstar *Taq* DNA polymerase (Qiagen, Shanghai, China) and water to 25 μL. mPCR was performed in single tube at 95°C for 15 min, followed by 35 cycles of 94°C for 30 sec, 60°C for 30 sec and 72°C for 1 min, finally by 72°C for 10 min. The development of RLB hybridization assay was described in previous studies [14].

#### Analytical specificity and sensitivity for mPCR/RLB

Extraction of DNA and adjustment of DNA concentration were performed with same operations as RT-PCR. In addition, serial ten-fold dilutions (starting at 10^5^ CFU/mL) of suspensions of cultures of *Escherichia coli* ATCC 25922 and *Streptococcus pneumoniae* SSI 14 were prepared in physiological saline.

### Statistical analysis

Statistical analyses were performed using the SPSS 19.0 software. Chi squared test was used to compare to the sensitivity of identified pathogens by assay and CSF cultures. A *P*-value of < 0.05 (2-tailed) was considered as significance.

## Results

### General clinical characteristics

56 infants were enrolled in the study. The written informed consent was obtained from the parents of all participants. The clinical characteristics of the 56 infants are shown in Table 4.The mean gestational age (GA) of infants was 38 weeks. The mean birth weight was 3.10 kg and 64% of them were male. The majority (73%) of the infants presented clinical symptoms after 1 week of life. Most (91%) of the infants had fever. Thirty-six infants (68%) had been treated with antimicrobials before hospitalization.

**Table 4:** **Characteristics of 56 neonatal infants**

Gestational age (week)	
<37	5 (9%)
37-42	51 (91%)
**Gender**	
Male	36 (64%)
Female	20 (36%)
**Onset**	
Early (<1 week)	15 (27)
Late (>1 week)	41 (73)
**Antibiotics before hospitalization**	
Yes	36 (64%)
No	20 (36%)
**Syptoms**	
fever	51 (91%)
Jaundice	23 (41%)
convulsion	10 (18%)
**Signs**	
Bulging fontanelle	21 (38%)
Limb muscle tension change	14 (25%)

Results of laboratory test of the 56 infants are shown Table 5. The initial median WBC was 18.03 × 10^9^/L, and 34 of the infants (61%) had leukocytosis with > 15 × 10^9^/L. CRP was elevated in 21cases (>8 mg/L).

**Table 5:** **Laboratory data of 56 neonatal infants**

Characteristics	All patients (n = 56)
**Blood**	
**Peripheral white cell count**	
(×10^9^/l)	22 (39%)
0-	34 (61%)
15-	
**Serum C-reaction protein (mg/l)**	
0-8	35 (63%)
>8	21 (38%))
**CSF**	
**WBC count (** **×10** ^**6**^ **/L** **)**	
0-	10 (18%)
21-	18 (32%)
>100	28 (50%)
**Glucose (mmol/l)**	
0-2.2	34 (61%)
>2.2	22 (39%)
**Protein (mg/l)**	
204-1000	26 (46%)
1001-2000	21 (38%)
>2000	9 (16%)
**CSF culture positive**	5 (9%)

### Clinical microbiology

Five CSF (9%) bacterial cultures were positive: two *L. monocytogenes*, one each of *S. pneumoniae*, *E. faecalis*, and *S. epidermidis,* respectively. Bacteria were isolated from blood cultures of nine patients (16%).

### Primers and probes

Target genes for each species, primer/probe sequences, and specificities, locations within target genes, numbered base positions and melting temperatures (*T*m) are shown in Table 6.

**Table 6:** **Primers and probes used in mPCR/RLB assay.**

Specificity	Primer/probe	Target	***T***m (°C)	GenBank accession No.	Primer and probe sequence (5′-3′)	Application product (bp)
*S. aureus*	SanucSb	*nuc*	65.68	V01281	GCG ATT GAT GGT GAT ACG GTT	278
*S. aureus*	SanucAb	*nuc*	69.12	V01281	AGC CAA GCC TTG ACG AAC TAA AGC	
*S. aureus*	SanucSp	*nuc*	61.06	V01281	GAT GGA AAA ATG GTA AAC GAA G	
*S. aureus*	SanucAp	*nuc*	61.36	V01281	CAT TGG TTG ACC TTT GTA CAT TAA	
*S. pneumoniae*	SpplySb	*ply*	67.47	M17717	CCC ACT CTT CTT GCG GTT GA	208
*S. pneumoniae*	SpplyAb	*ply*	61.68	M17717	TGA GCC GTT ATT TTT TCA TAC TG	
*S. pneumoniae*	SpplySp	*ply*	65.44	M17717	CCC AGC AAT TCA AGT GTT CG	
*S. pneumoniae*	SpplyAp	*ply*	65.49	M17717	CCA CTT GGA GAA AGC TAT CGC T	
*L.monocytogenes*	LmhlySb	*hly*	67.37	M24199	CAT GGC ACC ACC AGC ATC T	135
*L.monocytogenes*	LmhlyAb	*hly*	63.8	M24199	CAC TGC ATC TCC GTG GTA TAC TAA	
*L.monocytogenes*	LmhlySp	*hly*	68.2	M24199	GAA AAG AAA CAC GCG GAT GAA ATC	
*L.monocytogenes*	LmhlyAp	*hly*	65.33	M24199	TGG CGT CTT AGG ACT TGC AG	
*S. agalactiae*	GBScfbSb	*cfb*	59.53	X72754	ATG ATG TAT CTA TCT GGA ACT CTA GTG	259
*S. agalactiae*	GBScfbAb	*cfb*	60.48	X72754	CGC AAT GAA GTC TTT AAT TTT TC	
*S. agalactiae*	GBScfbSp	*cfb*	59.74	X72754	ATC AAA GAT AAT GTT CAG GGA AC	
*S. agalactiae*	GBScfbAp	*cfb*	58.55	X72754	TAC TTC TAA TAC AGC TGG TGA AAA	
*N. meningitidis*	NmctrASb	*ctrA*	66.14	AF520909	GCT GCG GTA GGT GGT TCA A	110
*N. meningitidis*	NmctrAAb	*ctrA*	66.36	AF5209	TTG TCG CGG ATT TGC AAC TA	
*N. meningitidis*	NmctrASp	*ctrA*	64.1	AF5209	ACG AAC TGT TGC CTT GGA AG	
*N. meningitidis*	NmctrAAp	*ctrA*	63.76	AF5209	ATT GCC ACG TGT CAG CTG	
*H. influenzae*	HigyrBSb	*gyrB*	62.57	U32738	GAA GCA CAG TCA TAA TAA CTT CTG CT	233
*H. influenzae*	HigyrBAb	*gyrB*	63.68	U32738	AGC GTC CTG GTA TGT ATA TCG G	
*H. influenzae*	HigyrBSp	*gyrB*	62.96	U32738	TTG CAC CGA TAC AGA ATT ATC ATC	
*H. influenzae*	HigyrBAp	*gyrB*	63.57	U32738	CGG GAT TCC TGT GGA TAT TC	
*E.coli*	Ecoli16SSb	16SrRNA	65.74	J01859	ATG CCG CGT GTA TCA AGA A	93
*E.coli*	Ecoli16SAb	16SrRNA	68.03	J01859	TAA CGT CAA TGA GCA A	
*E.coli*	Ecoli16SSp	16SrRNA	65.93	J01859	GGG GAG GAA GGG AGT AAA GT	
*E.coli*	Ecoli16SAp	16SrRNA	63.71	J01859	AGT ACT TTA CAA CCC GAA GGC	

### Results of the RT-PCR and mPCR/RLB analysis

**Figure 1:**
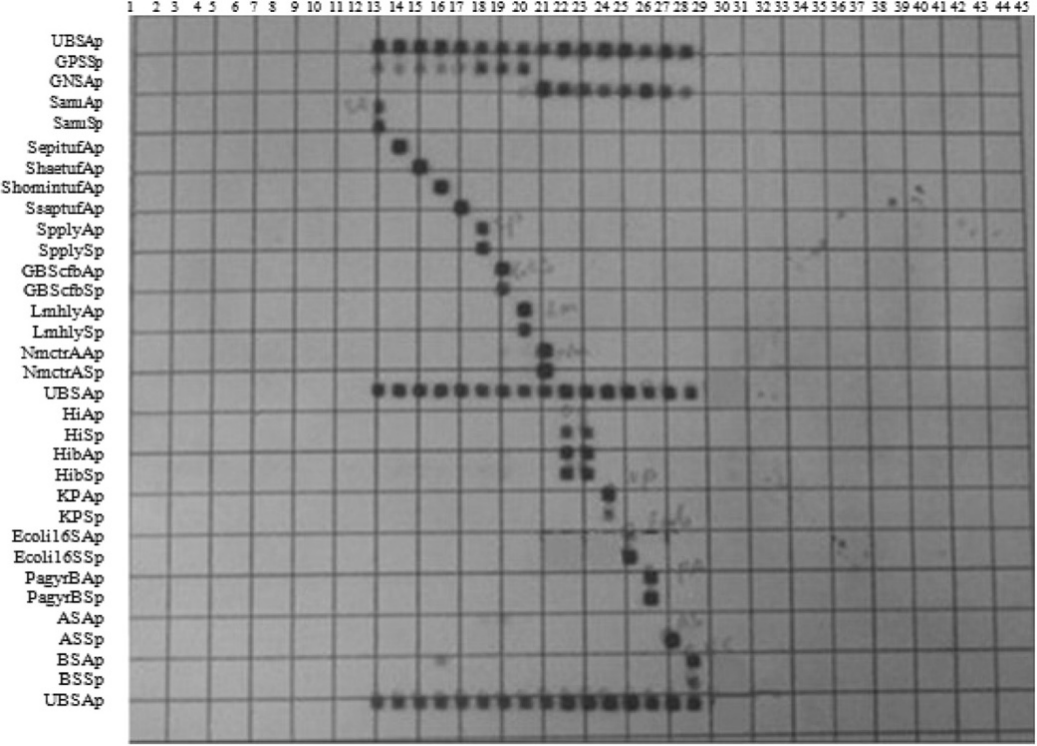
**Detection of 16 standard strains using the mPCR/RLB assay.**

The criteria for positive diagnostic hybridization was that at least one species-specific oligonucleotide probe gave a positive signal. Detection limits of the RT-PCR analysis varied among the 7 reference strains, from 2 to 200 fg of genomic DNA and mPCR/RLB from 50 to 500 fg of genomic DNA (Table 1). The sensitivity of the assay was from 0.3 to 91 cfu/μL for RT-PCR assay, and from 15 to 255 cfu/μL for mPCR/RLB assay (Table 1). None of the species-specific probes cross-reacted with any non-target species among the reference strains or clinical isolates (Figure 1 Detection of 16 standard strains using the mPCR/RLB assay).

### Detection of potential pathogens in CSF by RT-PCR and mPCR/RLB assays

Positive RT-PCR findings were detected in 25 of 56 CSF, including *E. coli* (10), *S. aureus* (7), *S. pneumonia* (3), *L. monocytogenes* (2), group B streptococci (2), and *N. meningitidis* (1).

16 CSF specimens of 56 cases were identified with positive mPCR/RLB, which consisted of *E. coli* (6), *S. aureus* (2), *L. monocytogenes* (2), group B streptococci (2), *S. pneumonia* (1), and *N. meningitides* (1). Two positive results by this assay required further analysis to be identified as S. epidermidis and E. faecalis.

### Comparison of RT-PCR assay with mPCR/RLB assay and CSF bacterial cultures

Overall, there were 28 cases with positive finding with these three test methods (bacterial culture, RT-PCR mPCR), indicating presence of pathogens. In one case, *S. pneumonia* was positive by culture only and negative by assay analysis. In two other cases, culture and mPCR/RLB were positive (*S. epidermidis* and *E. faecalis*), but the RT-PCR assay was negative. Biostatistics results are shown in Tables 7, 8 and 9. In brief, both RT-PCR and mPCR/RLB assays were more sensitive than bacterial culture in identification of pathogens, (*p* < 0.05), in addition, RT-PCR is more sensitive than mPCR/RLB assays (*p* < 0.05).

**Table 7:** **Comparison of Results of RT-PCR and mPCR/RLB Clinical Specimens**

		RT-PCR (N = 56)		Total
		+	-	
mPCR/RLB	+	14 (25.0%)	2 (3.6%)	16 (28.6%)
	-	11 (19.6%)	29 (51.8%)	40 (71.4%)
Total		25 (44.6%)	31 (55.4%)	56

**Table 8:** **Comparison of Results of RT-PCR and cultures**

		Cultures(N = 56)		Total
		+	-	
RT-PCR	+	2 (3.5%)	23 (41.1%)	25(44.6%)
	-	3 (5.4%)	28 (50.0%)	31 (55.4%)
Total		5(8.9%)	51(91.1%)	56

*χ* ^*2*^ = 13.885, *P* < 0.05.

**Table 9:** **Comparison of Results of mPCR/RLB and cultures**

		Cultures (N = 56)		Total
		+	-	
mPCR/RLB	+	4 (80.0%)	12 (23.5%)	16 (28.6%)
	-	1 (20.0%)	39 (76.5%)	40 (71.4%)
Total		5 (8.9%)	51 (91.1%)	56

*χ* ^*2*^ = 7.092, *P* < 0.05

## Discussion

It is critical important for rapid and specific identification of the causative agent in CSF and decision of optimal therapy in the clinical management of neonatal bacterial meningitis. CSF culture is routine laboratory tool and current gold standard for the diagnosis of neonatal bacterial meningitis in clinical practice. However, there are only small amount of positive CSF culture in the samples of neonatal bacterial meningitis [22]. Therefore, it would be diagnostic dilemma for the patients with negative CSF culture. Another disadvantage of CSF culture is that it needs up to 72 h for final identification.

Molecular methods, including RT-PCR and mPCR/RLB, do not depend on the presence of viable or growing bacteria, and thus are suitable to the detection of pathogens that cannot be cultured readily by routine methods, or that have been partially killed by exposure to antibiotics [6]. Our study shows RT-PCR and mPCR could be used for the identification of usual pathogens that cause meningitis in the newborn period. Both RT-PCR and mPCR/RLB are more rapid than culture [23]; RT-PCR can generally be completed two to three hours, and seven hours for mPCR/RLB. The consumable cost of RT-PCR and mPCR/RLB (U$ 20/specimen and U$ 7/specimen respectively) is more expensive than the culture with which cost of U$ 2/specimen.

mPCR/RLB assay, a molecular diagnostic tool, is based on the use of primers and probes that recognize conserved species-specific sequences of bacterial genes encoding essential molecules [17]. In this study, none of the species-specific probes cross-reacted with any non-target species among the reference strains or clinical isolates suggested its high specificity because using two probes for each target. Analysis of amplicons in the mPCR/RLB assay is more sensitive and faster than cultures, and 10^1^ to 10^2^ times more sensitive than common PCR [17]. A particular advantage of mPCR/ RLB is that the membranes can also be stripped and re-used up to 20 times without substantial loss of sensitivity [9]. The mPCR/ RLB method is potentially suitable for use with large numbers of specimens – like retrospective investigation and epidemiological surveillance, as it can analyze 43 clinical samples simultaneously.

In this study, 38 (68%) patients had been treated with antimicrobials before hospitalization, which could contributed to the low yield from CSF 6 (11%) and blood 9 (16%) cultures. At least one pathogen was identified in 16 (29%) of patients by using of mPCR/RLB and 25 (45%) by RT-PCR, respectively. This indicates that mPCR/RLB and RT-PCR (in particular) is significantly more sensitive than culture.

In this study, there was better correlation between culture and mPCR/RLB than the RT-PCR assay. RT-PCR failed to identify some specimens (*S. epidermidis* and *E. faecalis*) that were positive by culture and mPCR/RLB, which may be related with lack of corresponding RT-PCR primers and probes. In one case, mPCR/RLB did not identify S. pneumoniae, which grew on culture. This result may reflect inappropriate long term stored specimens (as a retrospective study), and/or the presence of mutations in the target regions of probes. The agreement between the two molecular methods was good. Overall, RT-PCR was relatively easy to perform and more sensitive than mPCR/RLB, suggesting that it is a useful tool for the diagnosis of bacterial meningitis. This is thought to be due to either (a) the presence of mutations in the target regions of probes or (b) competition among the 7 primer pairs in mPCR/RLB.

In this study, the most common pathogen was *Escherichia coli*, followed by *Staphylococcus aureus*, which is similar to the result reported by Airede [23].

Our study demonstrates that the RT-PCR and mPCR/RLB have the potential to identify pathogens better than bacterial culture in the cases with bacterial meningitis. Further studies will use RT-PCR and mPCR/RLB in larger population with bacterial meningitis in future, especially for the cases with negative CSF culture or other bacterial pathogens.

## Conclusion

RT-PCR and mPCR/RLB assays are potentially useful and reliable tools for the identification of neonatal bacterial meningitis. Both methods were found to be much more sensitive than culture particularly in the current series in which 68% of subjects had prior exposure to antibiotics. They detected the presence of pathogens in CSF samples that yield negative culture results. Further studies are necessary to confirm their utility and efficacy in optimizing the diagnosis and treatment of bacterial meningitis in young children.
